# Network-wise surface-based morphometric insight into the cortical neural circuitry underlying irritability in adolescents

**DOI:** 10.1038/s41398-021-01710-2

**Published:** 2021-11-10

**Authors:** Sahil Bajaj, Karina S. Blair, Johannah Bashford-Largo, Ru Zhang, Avantika Mathur, Amanda Schwartz, Jaimie Elowsky, Matthew Dobbertin, Soonjo Hwang, Ellen Leibenluft, R. James R. Blair

**Affiliations:** 1 Multimodal Clinical Neuroimaging Laboratory (MCNL), Center for Neurobehavioral Research, Boys Town National Research Hospital, Boys Town, NE USA; 2 Program for Trauma and Anxiety (PTAC), Center for Neurobehavioral Research, Boys Town National Research Hospital, Boys Town, NE USA; 3 Department of Psychiatry, University of Nebraska Medical Center, Omaha, NE USA; 4 Section on Mood Dysregulation and Neuroscience, National Institute of Mental Health, National Institutes of Health, Bethesda, MD USA

**Keywords:** Human behaviour, Diagnostic markers

## Abstract

Previous studies examining structural brain correlates of irritability have taken a region-specific approach and have been relatively inconsistent. In a sample of adolescents with and without clinically impairing irritability, the current study examines: (i) cortical volume (CV) in canonical functional networks; (ii) the association between the CV of functional networks and severity of irritability; and (iii) the extent to which IQ mediates the association between structural abnormalities and severity of irritability. Structural MRI and IQ data were collected from 130 adolescents with high irritability (mean age = 15.54±1.83 years, 58 females, self-reported Affective Reactivity Index [ARI] ≥ 4) and 119 adolescents with low irritability (mean age = 15.10±1.93 years, 39 females, self-reported ARI < 4). Subject-specific network-wise CV was estimated after parcellating the whole brain into 17 previously reported functional networks. Our Multivariate Analysis of Covariance (MANCOVA) revealed that adolescents with high irritability had significantly reduced CV of the bilateral control and default-mode networks (*p* < 0.05) relative to adolescents with low irritability. Multiple regression analyses showed a significant negative association between the control network CV and the severity of irritability. Mediation analysis showed that IQ partially mediated the association between the control network CV and the severity of irritability. Follow-up analysis on subcortical volume (SCV) showed that adolescents with high irritability had reduced bilateral SCV within the amygdala relative to adolescents with low irritability. Reduced CV within bilateral control and default networks and reduced SCV within bilateral amygdala may represent core features of the pathophysiology of irritability. The current data also indicate the potential importance of a patient’s IQ in determining how pathophysiology related to the control network is expressed.

## Introduction

Irritability is the propensity to initiate an angry response to frustration, threat, and social provocation and can be defined as “mood (trait) and behavioral (reactive state) dysregulation” that results in a propensity to react and respond angrily when an individual’s ability to attain a goal is blocked [1–3]. It can be clinically concerning when the individual’s expression of anger is high in relation to their peers [4, 5]. Prior work has been successful exploring the focal structural deficits associated with irritability but has limited our ability to better understand the network-wise (i.e., at a larger-scale) structural architecture of the brain underlying irritability. Notably, network-level architecture represents the organizational properties of distributed brain systems based on intrinsic functional connectivity patterns [6]. It’s still unclear how altered structural/cortical organizational properties of such distributed functional brain systems are associated with irritability in adolescents. The current study aims to determine the extent to which the network-wise cortical structure of adolescents with high irritability differs from adolescents with low irritability.

Functional neuroimaging work has relatively consistently related irritability to dysfunctions in both response control [7–9] and emotional responding [10, 11]. However, prior brain morphometry/structural studies have been less consistent with respect to: (i) the extent to which irritability is associated with greater cortical volume/cortical thickening [12, 13] or decreased grey matter volume/cortical thinning [14–17]; and (ii) the cortical regions implicated. Thus, among studies reporting that irritability is associated with greater grey matter volume/cortical thickening, one implicated the medial orbitofrontal and cingulum/cingulate cortices [12] and the other one implicated the superior frontal and temporal gyri, and the inferior parietal lobule [13]. Among studies reporting that irritability is associated with decreased grey matter volume/cortical thinning, two implicated the dorsolateral prefrontal cortex [15, 16] and two implicated the inferior frontal and temporal cortices [14, 17]. Thus, cortical alterations only within the frontal and temporal cortices appear to be consistent.

The first two goals of the current study were to determine network-wise cortical volume (CV) differences between adolescents with high irritability and adolescents with low irritability, and to further investigate associations between CV of identified networks (and their component regions) and irritability symptom severity. We measured CV from 17 cortical networks using one of the most advanced analytical methods, which does not over- or under-represent tissue according to the cortical convolutions [18]. Specifically, we used a network-based approach to parcellate the whole brain into 17 standard functional brain networks using Yeo’s atlas (see Supplementary Fig. 1 and Supplementary Information) [6]. To our knowledge, Yeo’s 17 network atlas is the only atlas that provides a detailed and finest network-wise cortical parcellation implemented in cutting-edge pipelines (e.g., in FreeSurfer toolbox) to quantify cortical morphometry. Prior work has used region-specific cortical parcellations in youth aged 9–10 years [19]; however, this is the first study where 17-network parcellation has been implemented in youth with irritability. While it is true this parcellation was originally developed from adult participants [6], subsequent work has demonstrated that 400 regional parcellation (that was assigned according to 17 network parcellation) reflects the network organization in youth aged 9–10 years [20]. Therefore, determining the CV of these networks and the extent to which they are atypical in individuals with high levels of irritability may improve the interpretability of findings in terms of their association with irritability. Because the primary focus of the current study is on the CV of 17 *cortical* networks, therefore, in our main analysis, we used *CV* as our primary parameter to quantify brain morphometry of each of the 17 *cortical* networks, whereas in follow-up analysis, we repeated our analysis and explored network-wise *thickness* and *surface area*, as well as *subcortical volume (SCV)* of the six main subcortical structures (i.e., thalamus, caudate, putamen, pallidum, hippocampus, and amygdala).

The third goal of this study was to determine the extent to which IQ mediates the association between the network-wise CV and irritability symptom severity. Previous work has shown strong associations between the cortical structure and IQ [21–23]. Specifically, IQ has been positively associated with a cortical thickness within the anterior-ventral prefrontal and superior frontal, superior parietal, and inferior and superior temporal cortices [22, 23] all regions implicated in structural MRI studies of irritability [14–17]. Higher IQ has also long been considered a protective factor against the development of mental illness (i.e., individuals with low IQ may be more vulnerable to mental illness) [24–26]. Higher IQ may facilitate the development of functions, such as executive functioning and internal locus of control [27–30] and as such may mediate the relationship between the cortical structure and mental illness. Specifically, the current study will test the extent to which IQ mediates the relationship between CV and irritability.

The goals of the current study were to (i) identify brain networks that show differences in CV between adolescents with high irritability and adolescents with low irritability; (ii) investigate the association between CV of identified brain networks (and their component regions) and irritability; and (iii) determine the extent to which IQ mediates the association between the altered network-wise cortical structure and greater levels of irritability. Given the prior neuroimaging work on irritability and dysfunctions in response control and emotional responding [7–11] and most of the prior structural MRI work showing decreased grey matter volume/cortical thinning associated with irritability [14–17], we predicted that irritability would be associated with reduced CV in networks hypothesized to underpin response control and emotional responding (i.e., the limbic, control, and default-mode networks). Specifically, we hypothesized that: (i) adolescents with high irritability would show lower CV within these networks compared to adolescents with low irritability; (ii) CV within these networks and their component regions would be negatively associated with irritability; and (iii) IQ would mediate the association between the altered cortical structure and irritability. In our follow-up exploratory analysis, we repeated our analysis with thickness and surface area as well as for SCV of six subcortical structures (i.e., thalamus, caudate, putamen, pallidum, hippocampus, and amygdala).

## Methods

### Participants

The current study included data collected from 249 youths between 11 and 19 years of age (*M*_*age*_ = 15.33±1.89 years, 97 females). Participants were recruited from a residential care facility at Boys Town National Research Hospital (BTNRH) and from the surrounding community. Participants recruited from the residential facility had been referred for behavioral and emotional problems. Participants were divided into high and low irritability groups based on their irritability scores and were matched across sex and age. Specifically, following guideline from previous work [31], those scoring 4 or greater on the Affective Reactivity Index (ARI) [32] were in the high irritability group (*N* = 130; 72 males; *M*_*age*_ = 15.54±1.83 years; age range: 11–19; ARI ≥ 4, *M*_*ARI*_ = 6.88 ± 2.52), while those scoring less than 4 were placed in the low irritability group (*N* = 119; 39 females; *M*_*age*_ = 15.10±1.93 years; age range: 11–19; ARI < 4; *M*_*ARI*_ = 0.84 ± 0.91). Consistent with that, receiver operating characteristic (ROC) analysis on the current sample showed that a cut-off value of 3.5 on self-reported ARI was optimal for indicating psychopathology; with both specificity and sensitivity of 100%. Here, all participants scoring ARI < 4 were recruited from the community and were basically a matched control group. However, the control group was referred as “low irritability group” in the current study because not all these participants scored 0 on the ARI scale but had ARI scores ranged between 0 and 4. Licensed and board-certified child and adolescent psychiatrists provided diagnoses following clinical interviews with the participants and their parents to adhere closely to common clinical practice. All participants and their parents provided written informed assent/consent prior to enrollment. The study protocol was approved by the Institutional Review Board at BTNRH. See Section 1 in Supplementary Material for details regarding exclusion/inclusion criteria.

### Data collection

#### Neuroanatomical data

Structural MRI data were collected using 3-Tesla MRI scanner located at BTNRH. Each participant was instructed repeatedly to try their best to minimize head movement during the entire scan. Custom-made padding was used to minimize head motion. Whole‐brain anatomical data for each participant were acquired using a 3D magnetization‐prepared rapid acquisition gradient echo (MPRAGE) sequence, which consisted of 176 axial slices (repetition time = 2200 ms, echo time = 2.48 ms, matrix size = 256 × 208, slice thickness = 1 mm, voxel resolution = 0.9 × 0.9 × 1 mm^3^, field of view (FOV) = 230 mm, and flip angle = 8^o^).

#### General intelligence (IQ)

The two-subset form [i.e., Full-Scale IQ-2 Subtests (FSIQ-2) from FSIQ-4] of the Wechsler Abbreviated Scale of Intelligence II (WASI-II) [33] was used to estimate IQ (mean IQ = 103.50±13.24) in the domains of matrix and vocabulary reasoning. The FSIQ-2 scores have a high-reliability coefficient (α = 0.93) in juvenile samples (age 6–16 years) [34] and a strong correlation of 0.94 with FSIQ-4 scores [35].

#### Self-reported affective reactivity index (ARI)

The ARI is a seven-item self-report questionnaire (six symptom items i.e., “I am easily annoyed by others”, “I often lose my temper”, “I stay angry for a long time”, “I am angry most of the time”, “I get angry frequently”, and “I lose my temper easily” and one function impairment item i.e., “Overall, my irritability causes me problems”) that assesses the youth’s irritability during the preceding 6 months [32]. Participants were asked to mark the box for “Not True”, “Somewhat True” or “Certainly True” corresponding to each item. Prior work has indicated that the ARI is a reliable (α = 0.88 in the US sample with a mean age of 12.90 ± 2.70 years; age range 6–17) and valid measure of irritability in youth [32].

#### Self-reported measures of other psychopathologies

Psychopathology was indexed via the: (i) *Conners ADHD scale* [36] to measure the severity of attention deficit/hyperactivity disorder symptoms; (ii) *Inventory of Callous-Unemotional Traits (ICU)* [37] to assess callous-unemotional traits (CU); (iii) *Mood and Feeling Questionnaire (MFQ)* [38] to assess depression symptomatology; and (iv) *Generalized Anxiety Disorder (GAD)* subscale using the Screen for Child Anxiety Related Disorders (SCARED) [39] to assess anxiety symptoms.

### Image preprocessing

Recent morphometry methods have allowed researchers to estimate several cortical measures, including CV, cortical thickness, and cortical surface area. As CV is the product of cortical thickness and cortical surface area, both cortical thickness and cortical surface area measurements influence CV measurements [40]. While the analysis of the CV (i.e., joint analysis of thickness and surface area) may be potentially more informative as it increases the power to simultaneously quantify the effects of thickness and surface area; however, analyzing thickness and surface area individually may also improve the specificity compared to CV. In our primary analysis, we used *CV* as our main parameter to quantify brain morphometry, whereas in follow-up analysis, we repeated our analysis and explored network-wise *thickness* and *surface area* as well. The “recon‐all” pipeline from the FreeSurfer toolbox (Version 6.0; https://surfer.nmr.mgh.harvard.edu) was used to process the structural brain images [41, 42] and for estimating CV, thickness, and surface area measures. Processing of structural images involved basic image preprocessing steps, including head motion‐correction, brain extraction (i.e., removal of nonbrain tissue), automated transformation to the standard MNI template space, volumetric segmentation into cortical and sub‐cortical matter, intensity correction, and parcellation of the cerebral cortex into gyral and sulcal matter [43]. The technical details of the preprocessing steps are documented in previous publications [41, 42, 44]. To inspect the preprocessing accuracy, standard quality control steps were performed. These steps included a careful visual inspection of raw structural images, skull‐stripped brain volumes, and pial surfaces.

### Data analysis

#### Outlier detection

Age, IQ, and ARI scores data were screened for outliers using SPSS 25 (https://www.ibm.com/analytics/spss-statistics-software). Any participants with a value (on either age, IQ, or ARI scores) more than 1.5 inter-quartile range above/below the upper/lower quartile were identified as outliers and were excluded from our analysis.

#### Demographics characteristics

Potential group differences in sex were examined via Chi-squared tests while those for age, IQ, and intracranial volume (ICV) were examined via two samples *t*-tests using SPSS 25.

#### Network-wise group differences in CV

The measure of CV (i.e., the amount of grey matter that lies between white-grey matter interface and pial matter) [40, 45] was evaluated separately for the left and the right hemisphere for each individual. Yeo’s Atlas [6] was used to estimate the CV of 17 functional brain networks for each hemisphere (for more details about these networks, see Supplementary Fig. 1 and Supplementary Information). For the between-group/main effect and between subject-effect analysis, hemispheric-wise CV data were compared between adolescents with high irritability and adolescents with low irritability using multivariate analysis of covariance (MANCOVA; with sex, age, IQ, and ICV as covariates) in SPSS 25. Only the networks that showed significant group differences (at *p* < 0.05) bilaterally (i.e., for both left and right hemispheres) were considered for further analysis. Multiple comparison correction was not performed across 17 networks because it is generally agreed that if MANCOVA shows a significant group effect, it is important to understand what components (in this case networks) are driving this group effect.

##### Statistical assumptions

Prior to conducting MANCOVA, CV data were tested for normality (using skewness and kurtosis) and homogeneity of the covariance (using Levene’s test of error variances) in SPSS 25. Data is considered to (a) be normal if skewness is ranged between −2 and +2 and kurtosis is between −7 and +7 [46], and (b) meet the assumption of homogeneity of the covariance if Levene’s test is not statistically significant. The current analysis showed that hemispheric-wise CV data followed a normal distribution with skewness ranged between −1 and +1 and kurtosis ranged between −1.1 and +1.1, and that the equality of variance assumption is true (*p* > 0.05) for 28 (out of 34) hemispheric-wise CV data points. Moreover, ARI data also followed a normal distribution with skewness and kurtosis magnitude of 0.61 and −0.69, respectively.

#### Associations between mean CV and irritability symptom severity

CV data were averaged over hemispheres for each identified network (i.e., the networks that bilaterally showed significant group differences). Dimensional analyses tested the association between hemispheric mean CV of the identified networks and irritability scores across the full sample. These analyses involved multiple regression with covariates (i.e., sex, age, IQ, and ICV) and mean CV of identified networks to potentially predict irritability scores.

#### Associations between mean region-specific CV and irritability symptom severity

CV data were averaged over hemispheres for all the component regions of each identified network that showed significant association with irritability. Here, component regions within each of the identified networks were extracted using *aparc.annot* (Desikan-Killany Atlas) [43], while the CV of these regions was extracted using the *mri_segstats* pipeline from FreeSurfer. Pearson’s partial correlation analyses (with sex, age, IQ, and ICV as covariates) were conducted to determine associations between hemispheric mean CV of component regions of the identified networks and irritability scores. Data points with Cook’s distance of more than four times the mean were considered as outliers and were excluded from the analysis. All the above analyses were conducted in MATLAB R2021a.

#### Mediation analysis: role of IQ in mediating the association between CV and irritability symptom severity

Separate standard mediation analyses (model 4) with 10,000 bootstrap samples were conducted using the Hayes PROCESS macro program [47] in SPSS 25 to examine the significance of indirect effects (at 95% confidence intervals) i.e., to determine the extent at which IQ mediates the association of (a) hemispheric mean CV of identified networks and (b) their component regions (i.e., networks/regions that showed significant association with irritability) with irritability. Because age and sex were not significant confounds, we did not include them in our mediation analysis. Data were standardized prior to conducting mediation analysis.

### Follow-up analyses

#### Inclusion of outliers. Our data were reanalyzed after retaining both the identified outliers

##### Potential confounds: impact of sex differences, age, IQ, ICV, psychopathologies, and prescribed medications

Sex differences in irritability scores were determined used two-sample *t* test. Correlation analyses were conducted to determine associations between ARI scores and age, IQ, measures of psychopathology (via Conners ADHD scale, ICU scale, MFQ scale, and GAD subscale), and current medication status (antipsychotic medications [*N* = 12], Selective Serotonin Reuptake Inhibitors [SSRIs; *N* = 22], and stimulants [*N* = 24]). Stepwise multiple regression analyses were conducted to evaluate whether potential confounds [i.e., sex, age, IQ, ICV, measures of psychopathology, prescribed medications (scored 1 for “yes” or 0 for “no”)] and hemispheric mean CV of identified networks significantly predicted irritability scores. To deal with multicollinearity, the regression analyses were performed for each measure of psychopathology separately.

##### Group Differences in cortical thickness and cortical surface area

All the main analyses (i.e., MANCOVA) performed for CV were repeated for cortical thickness and cortical surface area. For cortical thickness analysis, the potential covariates were sex, age, and IQ, whereas for cortical surface area analysis, the potential covariates were sex, age, IQ, and ICV.

##### Group differences in subcortical volume

All the main analyses (i.e., MANCOVA) performed for CV were repeated for subcortical volume for six subcortical areas i.e., thalamus, caudate, putamen, pallidum, hippocampus, and amygdala. For subcortical volume analysis, the potential covariates were sex, age, IQ, and ICV.

## Results

### Outlier detection

Based on age and ARI scores, none of the participants were identified as outliers. However, two participants (one from each irritability group) had IQ more than 1.5 interquartile range above the upper quartile and were, therefore, excluded from further analysis.

### Demographics characteristics

There were no group differences in sex (*χ*^*2*^ = 3.67, *p* = 0.06) and age (*t* (245) = −1.85, *p* = 0.065; *M*_*high irritability group*_ = 15.56, *SD* = 1.83; *M*_*low irritability group*_ =15.12, *SD* = 1.93). However, there were significant group differences in IQ (*t*(245) = 5.49, *p* < 0.001; *M*_*high irritability group*_ = 99.14, *SD* = 12.13; *M*_*low irritability group*_ =107.64, *SD* = 12.21) and ICV (*t*(245) = 2.45, *p* = 0.01; *M*_*high irritability group*_ = 1.47 × 10^6^ mm^3^, *SD* = 0.15 × 10^6^; *M*_*low irritability group*_ =1.52 × 10^6^ mm^3^, *SD* = 0.15 × 10^6^); see Table 1.

**Table 1:** Demographics.

Characteristics [*N* (%) or Mean (standard deviation)]	Adolescents with high irritability (*N* = 129)	Adolescents with low irritability (*N* = 118)	Group Differences (Two-sample *t* test/Chi-squared) (*p* value)	Overall Sample (*N* = 247)	Correlations with ARI^a^
Sex (Male/Female)	71/58	79/39	0.06	150/97	–
Ethnicity					
*Hispanic*	13 (10.1%)	6 (5.1%)		19 (7.7%)	
*Non-Hispanic*	108 (83.7%)	108 (91.5%)	0.12	216 (87.4%)	
*Unknown/not reported*	8 (6.2%)	4 (3.4%)		12 (4.9%)	
Race					–
*Native American*	2 (1.6%)	0 (0%)		2 (0.8%)	–
*Asian*	1 (0.8%)	1 (0.8%)		2 (0.8%)	
*Native Hawaiian or other Pacific Islander*	1 (0.8%)	0 (0%)	0.83	1 (0.4%)	–
*Black or African American*	15 (11.6%)	4 (3.4%)		19 (7.7%)	–
*White*	83 (64.3%)	103 (87.3%)		186 (75.3%)	–
*More than one race*	19 (14.7%)	9 (7.6%)		28 (11.3%)	–
*Unknown/not reported*	8 (6.2%)	1 (0.8%)		9 (3.6%)	–
Age	15.56 (1.83)	15.11 (1.93)	0.06	15.35 (1.88)	0.11
*Age range*	*10.50–18.25*	*12.11–18.88*			
IQ	99.14 (12.13)	107.64 (12.21)	<0.001**	103.20 (12.87)	−0.34**
* IQ range*	*70–132*	*79–135*			
ICV (x10^6^)	1.47 (0.15)	1.52 (0.15)	0.01*	1.49 (0.15)	−0.17*
* ICV range*	*1.07–1.88*	*1.17–1.96*			
ARI scores	6.89 (2.53)	0.84 (0.91)	<0.001**	4.00 (3.59)	–
* ARI range*	*4–12*	*0–3*			
ADHD scores	5.81 (6.71)	0.26 (0.93)	<0.001**	3.16 (5.61)	0.45**
* ADHD range*	*0–20*	*0–5*			
ICU scores	27.43 (8.84)	16.07 (6.10)	<0.001**	22.08 (9.54)	0.53**
* ICU range*	*11–51*	*3–32*			
MFQ scores	19.17 (13.58)	4.12 (4.10)	<0.001**	12.78 (12.97)	0.61**
* MFQ range*	*0–60*	*0–19*			
GAD scores	7.46 (4.74)	4.11 (2.99)	<0.001**	5.87 (4.34)	0.43**
* GAD range*	*0–18*	*0–15*			
Psychiatric Diagnosis					
*ADHD*	92 (71.3%)	–		92 (37.2%)	–
*MDD*	28 (21.7%)	–		28 (11.3%)	–
*GAD*	48 (37.2%)	–		48 (19.4%)	–
*SAD*	46 (35.7%)	–		46 (18.6%)	–
*PTSD*	25 (19.4%)	–		25 (10.1%)	–
*CD*	78 (60.5%)	–		78 (31.6%)	–
Medications					
*Antipsychotic*	12 (9.3%)	–		12 (4.9%)	0.17*
*SSRIs*	22 (17.1%)	–		22 (8.9%)	0.25**
*Stimulants*	24 (18.6%)	–		24 (9.7%)	0.24**

*ICV* intracranial volume (in mm^3^), *ARI* Affective Reactivity Index, *ADHD* Attention-Deficit/Hyperactivity.

Disorder; *ICU* Inventory of Callous-Unemotional Traits, *MFQ* Mood and Feeling Questionnaire, *GAD* Generalized.

Anxiety Disorder; *MDD* Major Depressive Disorder, *SAD* Social Anxiety Disorder, *PTSD* Posttraumatic Stress.

Disorder; *CD* Conduct Disorder, *SSRIs* Selective Serotonin Reuptake Inhibitors.

^a^Correlations between demographics of overall sample and ARI score; *p < 0.05; **p < 0.005.

### Network-wise group differences in CV

**Fig. 1:**
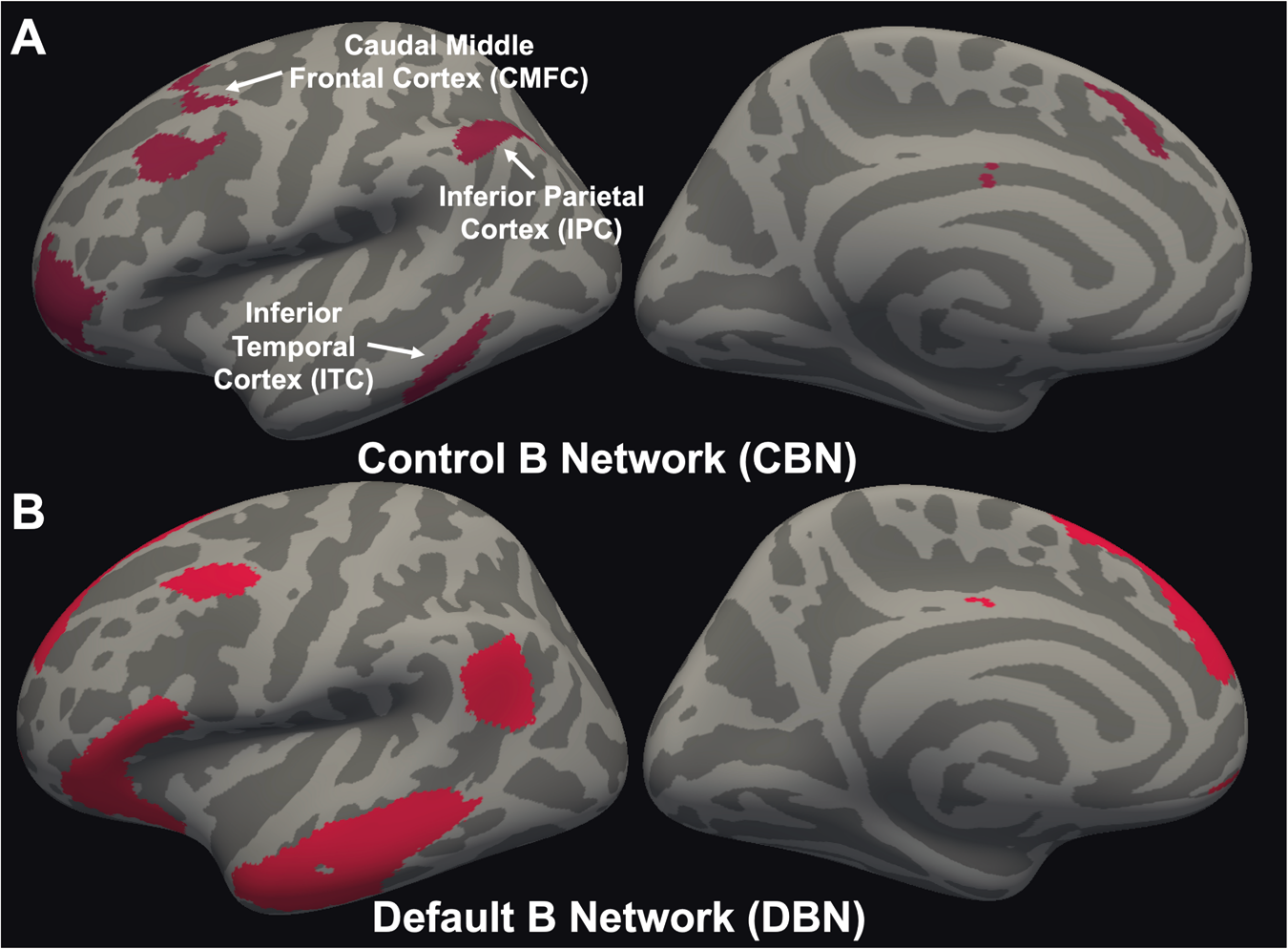
Cortical networks showing significant differences in CV. Hemispheric mean CV of the control B network (CBN) (**A**) and default B network (DBN) (**B**) were significantly different between adolescents with high irritability and adolescents with low irritability (adolescents with high irritability < adolescents with low irritability).

Our MANCOVA showed significant group differences in hemispheric-wise CV [*F* (34,208) = 1.55, *p* = 0.03; pη^2^ = 0.20; Wilk’s lambda = 0.80]. There were significant differences bilaterally in CV for the control B network (CBN; [*F* (1,241) = 6.79 & 8.59, *p* = 0.01 & 0.004, pη^2^ = 0.03 & 0.03, *respectively* for left and right hemisphere]; Fig. 1A) and default B network (DBN; [*F* (1,241) = 9.83 & 7.22, *p* = 0.002 & 0.01, pη^2^ = 0.04 & 0.03, *respectively* for left and right hemisphere]; Fig. 1B). For both networks, adolescents with high irritability showed lower CV than adolescents with low irritability. No bilateral significant differences in CV were seen in the other networks at *p* < 0.05 (see Table 2).

**Table 2:** Differences in Cortical Volume (CV): Adolescents with high irritability vs. adolescents with low irritability).

MANCOVA for CV (covariates: age, sex, IQ, and ICV)						
Networks (*N*)	*p* values		*F* value		Effect size: pη^2^	
	*LH*	*RH*	*LH*	*RH*	*LH*	*RH*
N1: Visual Peripheral (VPN)	0.04^a^	0.75	4.32	0.10	0.02	0.00
N2: Visual Central (VCN)	0.97	0.77	0.00	0.08	0.00	0.00
N3: Somatomotor A (SAN)	0.57	0.17	0.32	1.87	0.00	0.01
N4: Somatomotor B (SBN)	0.49	0.14	0.48	2.24	0.00	0.01
N5: Dorsal Attention A (DAAN)	0.29	0.008^a^	1.10	7.13	0.00	0.03
N6: Dorsal Attention B (DABN)	0.08	0.32	2.99	0.98	0.01	0.00
N7: Ventral Attention (VAN)	0.37	0.03^a^	0.80	5.05	0.00	0.02
N8: Salience (SN)	0.03^a^	0.43	4.60	0.61	0.02	0.00
N9: Limbic A (LAN)	0.02^a^	0.42	5.75	0.66	0.02	0.00
N10: Limbic B (LBN)	0.10	0.05	2.69	3.77	0.01	0.01
N11: Control C (CCN)	0.26	0.73	1.26	0.11	0.00	0.00
N12: Control A (CAN)	0.02^a^	0.56	5.11	0.33	0.02	0.00
N13: Control B (CBN)	**0.01**^a^	**0.004**^a^	**6.79**	**8.59**	**0.03**	**0.03**
N14: Default D (DDN)	0.91	0.006^a^	0.01	7.64	0.00	0.03
N15: Default C (DCN)	0.12	0.37	2.42	0.80	0.01	0.00
N16: Default A (DAN)	0.03^a^	0.05	5.01	3.78	0.02	0.01
N17: Default B (DBN)	**0.002**^a^	**0.01**^a^	**9.83**	**7.22**	**0.04**	**0.03**

*MANCOVA* Multivariate Analysis of Covariance, *ICV* intracranial volume.

*LH/RH* Left/Right Hemisphere.

^a^*p* < 0.05 (adolescents with high irritability < adolescents with low irritability).

Networks showing significant bilateral differences are bolded.

### Associations between mean CV and irritability symptom severity

Our stepwise multiple regression analysis revealed a significant regression equation for irritability scores [*F* (1,244) = 11.91; *p* = 0.001]. Adjusted *R*^2^ was 0.15. Significant predictors for irritability scores were CBN CV (standardized *B* = −0.21; *p* = 0.001) and IQ (standardized *B* = −0.31; *p* < 0.001). DBN CV, sex, age, and ICV were nonsignificant predictors for irritability scores (*B*_In_ = −0.07, 0.04, 0.02 & 0.03; *ps* = 0.56, 0.55, 0.69, & 0.68, respectively).

### Associations between mean region-specific CV and irritability symptom severity

Hemispheric-wise regions and a list of regions for which hemispheric mean CV was calculated for the CBN are summarized in Fig. 1A, Supplementary Table 1, and Supplementary Information. Within regions comprising the CBN, the caudal middle frontal cortex (CMFC) and inferior temporal cortex (ITC) showed significant negative partial associations between CV and irritability symptom severity (*r* = −0.16 & −0.13, *p* = 0.01 & 0.04, respectively; see Table 3). The inferior parietal cortex (IPC) also showed a strong trend of negative partial association between CV and irritability symptom severity (*r* = −0.13, *p* = 0.05; see Table 3).

**Table 3:** Pearson’s partial correlation (covariates: sex, age, IQ, and ICV) between region-specific mean cortical volume (CV) within the Control B Network (CBN) and irritability symptom severity.

Network	Regions	Correlation (r)	p-values	95% CI
**Control B Network (CBN)**	Superior Frontal Cortex (SFC)	0.10	0.14	[−0.03 0.22]
	**Caudal Middle Frontal Cortex (CMFC)**	**−0.16**	0.01*	[**−**0.28 **−**0.03]
	Rostral Middle Frontal Cortex (RMFC)	**−**0.07	0.25	[**−**0.20 0.05]
	Inferior Frontal Cortex (IFC)	0.11	0.08	[**−**0.01 0.24]
	**Inferior Temporal Cortex (ITC)**	**−0.13**	0.04*	[**−**0.25 **−**0.00]
	Posterior Cingulate Cortex (PCC)	0.02	0.79	[**−**0.11 0.14]
	**Inferior Parietal Cortex (IPC)**	**−0.13**	**0.05**	[**−**0.25 0.00]

*CI* Confidence Intervals; *p < 0.05.

Regions showing significant or trend towards significant correlation are bolded.

Bolded numbers in the table indicate significant associations.

### Mediation analysis: role of IQ in mediating the association between CV and irritability symptom severity

Two separate standard mediation analyses were conducted to determine the role of IQ in mediating the association between hemispheric mean CV of CBN and its component regions (i.e., CMFC, ITC, and IPC that showed significant association with irritability) and irritability symptom severity.

#### Network-specific CV and irritability symptom severity

Greater CBN CV was significantly associated with greater IQ (*r* = 0.16, *p* = 0.01) and lower levels of irritability (*r* = −0.25, *p* < 0.001). Greater IQ was also (independent of CBN CV) associated with lower levels of irritability (*r* = −0.31, *p* < 0.001). The mediation analysis revealed that while greater CBN CV was associated with lower levels of irritability (total effect, c = −0.25, *p* < 0.001), this association did not disappear once IQ was included as an “intervening” factor (direct effect, *c*′ = −0.20, *p* < 0.001). The bootstrap confidence interval for the indirect effect (ab = −0.05; [−0.09 −0.01] at 95% confidence interval) did not include zero. The percent mediation (P_M_) (i.e., percent of the total effect (c) accounted for by indirect effect (ab)) was 20%. Findings indicate that IQ partially accounted for the association between the CBN CV and irritability symptom severity.

#### Region-specific CV and irritability symptom severity

CMFC and IPC CVs were not significantly associated with IQ (*r* = 0.10 & 0.02, *p* = 0.10 & 0.77, respectively). Therefore, mediation analyses were not performed for CMFC and IPC. However, greater ITC CV was significantly associated with greater IQ (*r* = 0.13, *p* = 0.03) and lower levels of irritability (*r* = −0.20, *p* < 0.005). Greater IQ was also (independent of ITC CV) associated with lower levels of irritability (*r* = −0.32, *p* < 0.001). The mediation analysis revealed that while greater ITC CV was associated with lower levels of irritability (total effect, c = −0.20, *p* < 0.005), this association did not disappear once IQ was included as an “intervening” factor (direct effect, *c*′ = −0.15, *p* < 0.05). The bootstrap confidence interval for the indirect effect (ab = −0.04; [−0.08 −0.003] at 95% confidence interval) did not include zero. The percent mediation (P_M_) (i.e., percent of the total effect (c) accounted for by indirect effect (ab)) was 20%. Findings indicate that IQ partially accounted for the association between the ITC CV and irritability symptom severity.

**Fig. 2:**
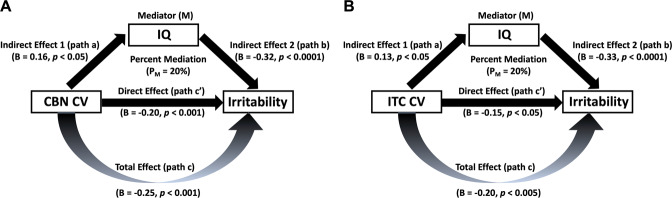
Role of IQ in mediating the association of hemispheric mean CV of the control B network (CBN) and its component region (i.e., inferior temporal cortex [ITC]) with irritability symptom severity. IQ partially accounted for the association of both CBN and ITC CVs with irritability symptom severity (Figs **A–B**, respectively).

Figure 2 illustrates the path model (model 4) used to test the mediation effect of IQ on the association between CBN and ITC CVs and irritability (Fig. 2A, B).

### Follow-up analyses

#### Inclusion of outliers

Reanalysis of our data after retaining both the identified outliers mostly mirrored our original results (see Supplementary section 2).

##### Potential confounds: impact of sex differences, age, IQ, ICV, psychopathologies, and prescribed medications

There were significant sex differences in irritability scores (*t* (245) = −2.79, *p* < 0.01; *M*_*males*_ = 3.49, *SD* = 3.34; *M*_*females*_ = 4.78, *SD* = 3.83). Irritability scores were not correlated with age (*r* = 0.11, *p* > 0.05) but were negatively correlated with IQ and ICV (*r* = −0.34 & −0.17, *ps* < 0.05; see Table 1). As expected, irritability scores were positively associated with measures of psychopathologies and prescribed medications (*r* = 0.17–0.61, *ps* < 0.05; see Table 1. Our stepwise multiple regression analyses revealed that CBN CV (standardized B ranges between −0.11 and −0.14, *ps ≤* 0.05) was still one of the significant predictors of irritability scores even after including demographics characteristics (sex, age, and IQ), ICV, measures of psychopathologies, and prescribed medications as independent variables in the model (see Section 3 in Supplementary Material).

##### Group differences in cortical thickness and cortical surface area

Our MANCOVA showed nonsignificant group differences in cortical thickness [*F* (34,209) = 0.68, *p* = 0.91; pη^2^ = 0.10] and cortical surface area [*F* (34,208) = 1.41, *p* = 0.08; pη^2^ = 0.19].

##### Group differences in SCV

Our MANCOVA showed significant group differences in SCV [*F* (12,230) = 2.24, *p* = 0.01; pη^2^ = 0.10]. No bilateral significant differences in SCV [*F* (1,247) = 0.00–5.58, *p* = 0.02–0.96, pη^2^ = 0.00–0.02] were seen in any of the subcortical structures at *p* < 0.05 (Table 4). However, there was a strong trend showing bilateral differences in SCV of the amygdala (left amygdala: *F* (1,247) = 3.76, *p* = 0.05 and right amygdala: *F* (1,247) = 4.12, *p* = 0.04). The amygdala also showed a significant negative partial association between hemispheric mean SCV and irritability symptom severity (*r* = −0.14, *p* = 0.03).

**Table 4:** Differences in Sub-Cortical Volume (SCV) (adolescents with high irritability vs. adolescents with low irritability).

MANCOVA for SCV (covariates: age, sex, IQ and ICV)						
Regions	*p* values		*F* value		Effect Size: pη^2^	
	*LH*	*RH*	*LH*	*RH*	*LH*	*RH*
Thalamus	0.02^a^	0.51	5.58	0.44	0.02	0.00
Caudate	0.30	0.96	1.07	0.00	0.00	0.00
Putamen	0.40	0.06	0.71	3.54	0.00	0.01
Pallidum	0.25	0.63	1.32	0.23	0.00	0.00
Hippocampus	0.06	0.45	3.58	0.56	0.01	0.00
Amygdala	0.05	0.04^a^	3.76	4.12	0.01	0.02

*MANCOVA* Multivariate Analysis of Covariance, *ICV* intracranial volume.

*LH/RH* Left/Right Hemisphere.

^a^*p* < 0.05 (adolescents with high irritability < adolescents with low irritability).

## Discussion

The goals of the current study were to identify the following: (i) networks showing CV differences in adolescents with high irritability relative to adolescents with low irritability; (ii) the extent to which network-wise CV and their component regions predicted irritability symptom severity; and (iii) the extent to which IQ mediated the association between CV of identified networks/regions and irritability symptom severity. Our data indicated that, relative to participants with low irritability, adolescents with high irritability had reduced CV within the control B and the default B networks (CBN and DBN) and reduced bilateral SCV within the amygdala. There was a negative association of CBN CV (and most of its regions, *specifically* the caudal middle frontal cortices, inferior temporal cortex, and inferior parietal cortex) and amygdala SCV with irritability symptom severity. Moreover, IQ partially mediated the association between the CBN CV and irritability symptom severity.

Regarding the direction of the cortical structure-irritability associations, previous studies have been inconsistent with respect to whether irritability is associated with greater cortical volume/cortical thickening [12, 13] or decreased grey matter volume/cortical thinning [14–17]. Our findings are clearly consistent with those studies associating irritability (or closely related constructs) with decreased grey matter volume/cortical thinning [14–17]. We note that these studies, as well as ours, differ from Dennis et al. 2019 and Pagliaccio et al. 2018 regarding participant age [12, 13]. Specifically, the two reports of associating irritability with greater cortical volume/cortical thickening involved relatively young participants (mean age = 11.5 years [12]; mean age = 4.47 years [13]). In contrast, the mean age of participants with irritability in the current study was 15.35 years, similar to that in Adleman et al. (14.2 years) [15], Chaarani et al. (14.5 years) [17], Gold et al. (14.6 years) [16], and Jirsaraie et al. (16.2 years) [14]. Moreover, prior work has indicated that mean cortical thickness decreases while mean cortical surface area increases with age (and CV is more closely associated with cortical surface area [40]) and both cortical thickness and cortical surface area follow a linear trend [48]. However, mean CV follows either a linear or a quadratic trend, depending on regions of interest [48]. Interestingly, overall, maxima or minima was observed at roughly 12 years of age when a quadratic function was fit to best illustrate the age-related changes [48]. The observed cut-off age of roughly 12 years here is approximately consistent with the mean age of 11.5 years in the study associating irritability with greater cortical volume/cortical thickening [12]. In short, we believe the current data (mean age = 15.35 years), together with previous work on different CV/thickness/surface area-age trajectories across different age groups, suggests that, for relatively older adolescents, irritability is associated with decreased grey matter volume/cortical thinning in relevant regions. Future research will benefit from longitudinal data collected across a wide age range in adolescents.

Consistent with our a priori hypotheses, there were significant group differences within the control and default mode networks (specifically, significantly decreased CBN and DBN volumes for the adolescents with high irritability as compared to adolescents with low irritability). While there were group differences in network volumes and IQ, IQ could not account for these differences—given the results of our stepwise multiple regression with IQ as a covariate. To our knowledge, these results are the first to show network-wise structural alterations (at a whole-brain level) associated with elevated irritability. Prior structural MRI studies have mostly reported region-specific structural alterations associated with irritability [12–14, 17]. Notably, our data are consistent with prior functional work suggesting that dysfunction in components of the CBN mediating inhibitory control is associated with increased irritability [7–9, 49]. In addition, some connectivity analyses of resting-state functional MRI data have examined associations of network-level connectivity (particularly in the default-mode network) with constructs that are closely related to irritability. Specifically, the negative mood state of anger has been associated with connectivity alterations within the default-mode network [50, 51]. Patients with bipolar or borderline personality disorders, which are characterized by high levels of anger, have shown both abnormal functional [52, 53] and structural organization (e.g., reduced grey matter) [54] within the default-mode network. Moreover, prior functional MRI work has associated irritability with atypical functioning in components of the default-mode network [10, 11]. Thus, despite the lack of prior network-wise structural MRI work on irritability, the current results are consistent with previous network-wise functional and structural MRI work, particularly on the default-mode network and constructs that are closely related to irritability (e.g., anger or bipolar and borderline personality disorder).

In contrast to our hypotheses, there were no significant group differences within the limbic network. Given strong empirical claims that irritability is associated with atypical emotional responding [3, 55], this was unexpected. Moreover, none of the subcortical structures showed bilateral group differences in subcortical volume. Future work will be needed to explore these issues further. Also, there were no significant group differences in cortical thickness and cortical surface area, but CV showed significant group differences. As mentioned before, both the cortical thickness and cortical surface area measurements influence CV measurements [40]. Therefore, we observed significant group differences in CV only as the joint analysis of thickness and surface area in terms of CV increases the power to simultaneously quantify the effects of thickness and surface area.

CV within the CBN, but not the DBN, was negatively associated with irritability symptom severity. With respect to specific anatomical regions within the CBN, there were significant negative associations between CV of the caudal middle frontal cortex, inferior temporal cortex, and the inferior parietal cortex and irritability (see Table 3). These findings were consistent with previous functional neuroimaging studies of irritability which have associated atypical recruitment of regions within the middle frontal cortex and inferior parietal lobule with irritability [49, 56]. In a review paper on the adult population, decreased grey matter volume/cortical thinning within the middle frontal gyrus and a large proportion of the temporal cortex has been found in patients with bipolar disorder [57]. In sum, prior functional and structural findings on irritability support the currently observed ‘region-specific’ associations of irritability.

In line with our predictions, IQ at-least partially mediated the relationship between pathophysiology and irritability. Notably, this mediation was true only for the relationship between CBN CV (and its component region ITC) and irritability. Given the cross-sectional nature of the current study, the mediation findings are purely based on correlation analyses and should not be interpreted or viewed as “causal” in nature. These results are consistent with our predictions generated from previous work indicating that IQ (i.e., the overall level of general cognitive and intellectual functioning, and faster processing speeds/lower reaction time) is inversely associated with the risk for the expression of a variety of psychiatric disorders, including ADHD [24], depression [58], and, importantly for the current study, anger as a response to stress [59]. We hypothesize that this reflects a role for IQ in fostering the development of executive function (e.g., working memory and internal locus of control) [27–30, 60] that reduces the probability that the individual will express an underlying neuro-biological risk factor in symptomatology. Prior work has shown that IQ/general intelligence was significantly associated with both—at-least 80% executive function indices [28, 61], as well as measures of locus of control [29, 62]. Both executive function and a more internal locus of control have also been associated with reduced psychological distress [30, 63] and aggression [64–66]. We assume that the negative association between CBN CV and irritability reflects poorer control over the behavioral expressions of irritability. As such, an increased ability to control behavior (executive function more generally) and an increased sense that the individual can control their behavior (locus of control) may protect the individual from developing the irritability symptom profile. Future work will be necessary to examine these speculations in more detail. Additionally, the cortical association area 36 that involves portions of the lateral ITC (and medial fusiform gyrus) has a strong influence on cognitive and visual recognition abilities, mostly assessed with general IQ testing [22]. The association of structural deficits within inferior temporal regions with both reduced cognitive/recognition abilities [22] and elevated levels of irritability [14] support the notion that IQ might be playing a mediating role between reduced CV within the ITC and irritability symptom severity. Moreover, temporal regions, by themselves, are not only crucial for affect regulation [67] but may also modulate aggression via activating cortical and subcortical emotional response system through its connections with the amygdala and hypothalamus [68]. In sum, structural alteration in such regions may be critical for elevated levels of irritability [14].

The current findings must be viewed considering the following three caveats. First, there were group differences in IQ and ICV. However, mitigating this concern, the effect of IQ and ICV was controlled for within our group analyses. Second, networks of interest were constrained only if there were bilateral significant differences in morphometric measures. Therefore, the hemispheric laterality was not analyzed in the current study. Third, the participants with high irritability showed significant psychiatric comorbidities and some were receiving psychiatric medications. However, the follow-up analyses that we conducted with psychiatric comorbidities and psychiatric medications as additional predictors in our multiple regression analyses mirrored our results. This suggested that psychiatric comorbidities and psychiatric medications did not confound the current results and that CBN CV was still one of the significant predictors of irritability.

In summary, we found significantly lower CV within the control and default-mode networks in adolescents with high irritability as compared to adolescents with low irritability. Moreover, control network CV showed a significant negative association with irritability. The association between control network CV and irritability was partially mediated by IQ. The current findings enhance our fundamental understanding of cortical structure and irritability at the level of large-scale brain networks that have been specified by considerable prior functional and structural MRI work [69–74], and further suggest core structural impairments related to the expression of irritability.

## Supplementary information

Supplementary Information

Supplementary Material

Supplementary Table

Supplementary Figure
