# Supplementary material for: Network-wise surface-based morphometric insight into the cortical neural circuitry underlying irritability in adolescents
Source: Transl Psychiatry. 2021 Nov 10;11:581. doi: 10.1038/s41398-021-01710-2 (PMC8581009; doi:10.1038/s41398-021-01710-2)
Supplement: Supplementary file 1 — Supplementary Information [file 41398_2021_1710_MOESM1_ESM.docx]

**Supplemental Information**

**Supplementary Figure and Table Legends**

*Supplemental Figure 1.* Whole Brain Parcellation into 17 Brain Networks. Left hemisphere (A): lateral and medial views; right hemisphere (B): lateral and medial views, and left and right hemispheres (C): dorsal view.

*Supplemental Table 1.* List of individual cortical regions within the control B network (CBN) and list of regions within the CBN for which hemispheric mean cortical volume (CV) was calculated.
