# Supplementary material for: Network-wise surface-based morphometric insight into the cortical neural circuitry underlying irritability in adolescents
Source: Transl Psychiatry. 2021 Nov 10;11:581. doi: 10.1038/s41398-021-01710-2 (PMC8581009; doi:10.1038/s41398-021-01710-2)
Supplement: Supplementary file 2 — Supplementary Material [file 41398_2021_1710_MOESM2_ESM.docx]

**Supplemental Material**

**Section 1: Exclusion/inclusion Criteria**

Exclusion criteria included IQ < 75 (Wechsler, 2011), pregnancy, non-psychiatric medical conditions that require the use of medication that may have psychotropic effects (e.g., steroids or beta blockers), current psychosis, pervasive developmental disorders, Tourette’s disorder, neurological disorders, presence of metallic objects in the body (e.g., metal plates, pacemakers, etc.), and claustrophobia. Current psychiatric conditions (other than psychotic disorders or pervasive developmental disorders) and use of psychotropic medications for psychiatric indications (e.g., antipsychotic medications, stimulants, or selective serotonin reuptake inhibitors [SSRI]) were not included in exclusion criteria. However, participants on stimulant medication were asked to withhold medication on the day of scanning.

**Section 2: Inclusion of Outliers**

We reanalyzed our data after retaining both the identified outliers. Reanalysis mirrored our original results:

**Demographics Characteristics**

There were no group differences in sex (*χ^2^* = 3.66, *p =* 0.06) and age (*t* (247) = -1.86, *p* = 0.06; *M_high irritability group_* = 15.54, *SD* = 1.83; *M_low irritability group_* =15.10, *SD* = 1.93). However, there were significant group differences in IQ (*t*(247) = 5.31, *p* < 0.001; *M_high irritability group_* = 99.45, *SD* = 12.61; *M_low irritability group_* =107.92, *SD* = 12.53) and ICV (*t*(247) = 2.54, *p* = 0.01; *M_high irritability group_* = 1.47 x 10^6^ mm^3^, *SD* = 0.15 x 10^6^; *M_low irritability group_* =1.52 x 10^6^ mm^3^, *SD* = 0.15 x 10^6^).

**Network-wise Group Differences in CV**

Our MANCOVA showed significant group differences in hemispheric-wise CV [*F* (34,210) = 1.54, *p* = 0.04; pη^2^ = 0.20; Wilk’s lambda = 0.80]. There were significant differences bilaterally in CV for the control B network (CBN; [*F* (1,243) = 6.09 & 8.10, *p =* 0.01 & 0.005, pη^2^ = 0.02 & 0.03, *respectively* for left and right hemisphere]) and default B network (DBN; [*F* (1,243) = 8.63 & 7.07, *p =* 0.004 & 0.01, pη^2^ = 0.03 & 0.03, *respectively* for left and right hemisphere]). In addition, there were significant differences bilaterally in CV for the default A network (DAN; [*F* (1,243) = 4.96 & 4.08, *p =* 0.03 & 0.04, pη^2^ = 0.02 & 0.02, *respectively* for left and right hemisphere]).

For all three identified networks, adolescents with high irritability showed lower CV than adolescents with low irritability. No bilateral significant differences in CV were seen in the other networks at *p* *<* 0.05.

**Associations between Mean CV and Irritability Symptom Severity**

Our stepwise multiple regression analysis revealed a significant regression equation for irritability scores [*F* (1,246) = 12.16; *p* = 0.001]. Adjusted R^2^ was 0.15. Significant predictors for irritability scores were CBN CV (standardized B = -0.21; *p* = 0.001) and IQ (standardized B = -0.30; *p <* 0.001). DBN CV, DAN CV, sex, age, and ICV were non-significant predictors for irritability scores (B_In_ = -0.08, -0.05, 0.04, 0.02 & 0.02; *ps* = 0.56, 0.70, 0.59, 0.74 & 0.82, respectively).

**Associations between Mean Region-specific CV and Irritability Symptom Severity**

Hemispheric-wise regions and a list of regions for which hemispheric mean CV was calculated for the CBN are summarized in Figure 1A, Supplemental Table 1, and Section 1 in Supplemental Material. Within regions comprising the CBN, the caudal middle frontal cortex (CMFC) showed significant partial association between CV and irritability symptom severity (*r =* -0.15, *p =* 0.02) (with sex, age, IQ and ICV as covariates, and after excluding data points with Cook's distance of more than four times the mean). The inferior parietal cortex (IPC) and the inferior frontal cortex also showed a trend of partial associations between CVs and irritability symptom severity (*r =* -0.12 & 0.12; *p =* 0.07 & 0.06). Interestingly, the inferior temporal cortex (ITC) CV was not significantly associated with irritability symptom severity (*r =* -0.10, *p =* 0.13).

**Mediation Analysis: *Role of IQ in Mediating the Association between CV and Irritability Symptom Severity***

Two separate standard mediation analyses (model 4) were conducted to determine the role of IQ in mediating the association between hemispheric mean CV of CBN (and its component region i.e., CMFC) that showed significant association with irritability) and irritability symptom severity. For mediation analysis, sex, age, and ICV were not added as covariates. Also, no data points were excluded based on Cook's distance.

***Network-specific CV and Irritability Symptom Severity***

Greater CBN CV was significantly associated with greater IQ (*r* = 0.16, *p* = 0.01) and lower levels of irritability (*r* = -0.21, *p* < 0.001). Greater IQ was also (independent of CBN CV) associated with lower levels of irritability (*r* = -0.30, *p* < 0.001). The mediation analysis revealed that while greater CBN CV was associated with lower levels of irritability (total effect, c = -0.26, *p* < 0.001), this association did not disappear once IQ was included as an "intervening" factor (direct effect, *c′* = -0.21, *p* < 0.001). The bootstrap confidence interval for the indirect effect (ab = -0.05; [-0.09 -0.01] at 95% confidence interval) did not include zero. The percent mediation (PM) (i.e., percent of the total effect (c) accounted for by indirect effect (ab)) was 19%. Findings indicate that IQ partially accounted for the association between CBN CV and irritability symptom severity.

***Region-specific CV and Irritability Symptom Severity***

CMFC CV was not significantly associated with IQ (*r* = 0.10, *p* = 0.10). Therefore, mediation analysis was not performed for CMFC. To further check if our original mediation results for the IPC and ITC were also consistent after including the two outliers, we again performed mediation analyses for the IPC and ITC CVs after including both the outliers. Consistent with our original results, IPC CV was not significantly associated with IQ (*r* = 0.02, *p* = 0.77). Therefore, mediation analysis was not performed for IPC. However, greater ITC CV was significantly associated with greater IQ (*r* = 0.13, *p* = 0.03) and lower levels of irritability (*r* = -0.15, *p* = 0.01). Greater IQ was also (independent of ITC CV) associated with lower levels of irritability (*r* = -0.33, *p* < 0.001). The mediation analysis revealed that while greater ITC CV was associated with lower levels of irritability (total effect, c = -0.20, *p* < 0.005), this association did not disappear once IQ was included as an "intervening" factor (direct effect, *c′* = -0.15, *p* < 0.05). The bootstrap confidence interval for the indirect effect (ab = -0.04; [-0.08 -0.004] at 95% confidence interval) did not include zero. The percent mediation (PM) (i.e., percent of the total effect (c) accounted for by indirect effect (ab)) was 20%. Consistent with original findings, here the findings again indicate that IQ partially accounted for the association between ITC CV and irritability symptom severity.

**Section 3: Multiple Regression Analyses for Irritability Symptom Severity**

First regression analysis included demographic characteristics (i.e., sex, age, and IQ), ICV, scores on Conners ADHD scale, prescribed medications, and CBN CV as independent variables. Our analyses revealed significant regression equation for irritability scores [*F* (1,242) = 6.56, *p* = 0.01, adjusted R^2^ = 0.34]. Significant predictors for irritability scores were IQ (standardized B = -0.26; *p <* 0.001), ADHD scores (standardized B = 0.39; *p <* 0.001), SSRI (standardized B = 0.22; *p* < 0.001), and CBN CV (standardized B = -0.14; *p* = 0.01).

Second regression analysis included demographic characteristics, ICV, scores on ICU scale, prescribed medications, and CBN CV as independent variables. Our analyses revealed significant regression equation for irritability scores [*F* (1,235) = 7.51, *p* = 0.01, adjusted R^2^ = 0.38]. Significant predictors for irritability scores were IQ (standardized B = -0.22; *p <* 0.001), ICU scores (standardized B = 0.45; *p <* 0.001), SSRI (standardized B = 0.17; *p* < 0.005), and CBN CV (standardized B = -0.14; *p* = 0.01).

Third regression analysis included demographic characteristics, ICV, scores on MFQ scale, prescribed medications, and CBN CV as independent variables. Our analyses revealed significant regression equation for irritability scores [*F* (1,187) = 3.76, *p* = 0.05, adjusted R^2^ = 0.45]. Significant predictors for irritability scores were IQ (standardized B = -0.20; *p <* 0.001), MFQ scores (standardized B = 0.51; *p <* 0.001), SSRI (standardized B = 0.12; *p* = 0.03), stimulant (standardized B = 0.13; *p* = 0.03), and CBN CV (standardized B = -0.11; *p* = 0.05).

Fourth regression analysis included demographic characteristics, ICV, scores on GAD scale, prescribed medications, and CBN CV as independent variables. Our analyses revealed significant regression equation for irritability scores [*F* (1,232) = 3.92, *p* = 0.05, adjusted R^2^ = 0.34]. Significant predictors for irritability scores were IQ (standardized B = -0.27; *p <* 0.001), GAD scores (standardized B = 0.36; *p <* 0.001), SSRI (standardized B = 0.11; *p* = 0.04), stimulant (standardized B = 0.22; *p* < 0.001), and CBN CV (standardized B = -0.11; *p* = 0.05).

**References**

**Wechsler D** (2011) Wechsler Abbreviated Scale of Intelligence–Second Edition (WASI-II). San Antonio, TX: NCS Pearson.
