# Supplementary material for: Network-wise surface-based morphometric insight into the cortical neural circuitry underlying irritability in adolescents
Source: Transl Psychiatry. 2021 Nov 10;11:581. doi: 10.1038/s41398-021-01710-2 (PMC8581009; doi:10.1038/s41398-021-01710-2)
Supplement: Supplementary file 3 — Supplementary Table [file 41398_2021_1710_MOESM3_ESM.docx]

**Supplementary Table 1**

| **Networks** | **Anatomical regions/components** |
| --- | --- |
| **Control B Network (CBN)** | **LH:** Superior Frontal Cortex (SFC), Caudal Middle Frontal Cortex (CMFC) extended to Superior Frontal Cortex and Rostral Middle Frontal Cortex, Rostral Middle Frontal Cortex (RMFC) extended to Inferior Frontal Cortex (Pars Orbitalis and Lateral Orbitofrontal Cortex), Inferior Temporal Cortex (ITC) extended to Middle Temporal Cortex, Posterior Cingulate Cortex (PCC), and Inferior Parietal Cortex (IPC) extended to Supramarginal Gyrus  **RH:** Superior Frontal Cortex (SFC), Caudal Middle Frontal Cortex (CMFC) extended to Superior and Rostral Middle Frontal Cortex, Rostral Middle Frontal Cortex (RMFC), Rostral Middle Frontal Cortex (RMFC) extended to Inferior Frontal Cortex (Pars Orbitalis and Lateral Orbitofrontal Cortex), Inferior Frontal Cortex (IFC) (Pars Opercularis and Pars Triangularis), Inferior Temporal Cortex (ITC) extended to Middle Temporal Cortex, and Inferior Parietal Cortex (IPC) |
| **Hemispheric Mean CBN** | SFC, CMFC, RMFC, IFC, ITC, PCC, and IPC |

*LH/RH: Left/Right Hemisphere*
